# Impacts of traditional food consumption advisories: Compliance, changes in diet and loss of confidence in traditional foods

**DOI:** 10.1186/1476-069X-10-55

**Published:** 2011-06-08

**Authors:** Claire McAuley, Loren D Knopper

**Affiliations:** 1 Stantec Consulting Ltd., 10160 - 112 Street, Edmonton AB T5K 2L6, Canada; 2 Stantec Consulting Ltd., 200 - 2781 Lancaster Road, Ottawa ON K1B 1A7, Canada

## Abstract

**Background:**

Food consumption advisories are often posted when industrial activities are expected to affect the quality and availability of traditional foods used by First Nations. We were recently involved in a project and asked to summarize details regarding the impacts of traditional food consumption advisories with respect to compliance, broader changes in diet and loss of confidence in traditional foods by people.

**Methods:**

Our review was not conducted as a formal systematic comprehensive review; rather, we focused on primary and grey literature presenting academic, health practitioner and First Nations viewpoints on the topic available from literature databases (i.e., PubMed, Web of Knowledge^SM^) as well as the internet search engine Google. Some information came from personal communications.

**Results:**

Our overview suggests that when communicated effectively and clearly, and when community members are involved in the process, consumption advisories can result in a decrease in contaminant load in people. On the other hand, consumption advisories can lead to cultural loss and have been linked to a certain amount of social, psychological, nutritional, economic and lifestyle disruption. In some cases, communities have decided to ignore consumption advisories opting to continue with traditional lifestyles believing that the benefits of doing so outweigh the risk of following advisories.

**Conclusions:**

We identified that there are both positive and negative aspects to the issuance of traditional food consumption advisories. A number of variables need to be recognized during the development and implementation of advisories in order to ensure a balance between human health, maintenance of cultures and industrial activity.

## Background

Industrial development can affect the quality and availability of traditional or country foods used by First Nations. Normal industrial operations (e.g., possible deposition of heavy metals or organics from mining activity, creation of methyl mercury from inundation of land from flooding or river damming for hydroelectric projects) and possible upset conditions created by accidents or malfunctions (e.g., possible releases of chemicals to the environment through spills, leaks or industrial fires) can affect food quality. In other words, foods can become unpalatable because of poor taste (e.g., often the case with fish) or possibly unhealthy because of chemical concentrations (e.g., as can be the case with vegetation used for consumption or as medicinal teas). As a result, food consumption advisories are often posted when industrial activities (and in some cases when natural parameters are known to affect food quality (i.e., Health Canada has posted fish consumption advisories for commercially sold fish due to methylymercury biomagnification in aquatic food webs) are expected to be associated with these adverse effects.

We were recently involved in a project that has the potential to affect food quality as a result of anthropogenic activity and asked to summarize details regarding the impacts of traditional food consumption advisories with respect to compliance, broader changes in diet and loss of confidence in traditional foods by people. To do this, we focused on primary and grey literature (available in literature databases (i.e., PubMed, Web of Knowledge^SM^) as well as the internet search engine Google) presenting academic, health practitioner and First Nations viewpoints on these topics. Some information for our summary came from personal communication with researchers in the field. We did not conduct a formal systematic comprehensive review. Our overview suggests that when communicated effectively and clearly, and when community members are involved in the process, consumption advisories can result in a decrease in contaminant load in people. On the other hand, traditional food consumption advisories can lead to cultural loss and have been linked to a certain amount of social, psychological, nutritional, economic and lifestyle disruption. With the consent of our client we thought it appropriate to submit this report as a commentary piece in an open access journal with multi-disciplinary readership so the information would be accessible to a larger audience that may be interested in the subject.

### Impacts of Traditional Food Consumption Advisories with Respect to Compliance

Studies conducted on polychlorinated biphenyl (PCB) contamination in fish for the Mohawk Nation of Akwesasne from 1995 - 1999 provide valuable information on the role of First Nation participants in exposure assessments through both communication and participation. Studies conducted in 1995 (to assess exposure to PCBs for nursing Mohawk women), in 1996 (to assess dietary, residential and occupational exposures to PCBs and DDE and body burden of Mohawk men) and in 1998 (to assess the relationship between the consumption of contaminated fish and concentrations of PCBs and PCB congeners in the milk of nursing mothers at Akwesasne) yielded results showing significantly higher body burdens, blood levels and milk levels of organochlorines than in Caucasian control populations [1]. The study found that fish were contaminated with PCBs and that there was a higher rate of fish consumption among the Mohawk population than compared to their Caucasian control populations [1]. Fish consumption advisories were established as a result of the studies and have effectively reduced the body burdens of PCBs for Akwesasne community members. The effectiveness of these advisories was attributed to the involvement of Native researchers in the studies as an integral part of the research team. First Nation researchers administered interviews, developed the questionnaire and identified and recruited participants. It was acknowledged by the researchers that the success of the program was due to the Mohawk community's willingness to cooperate with the sampling efforts which was attributable to the field staff being of Mohawk heritage and the genuine concern of the Mohawk people for their environment [1].

As part of the Mercury Agreement, signed by the Government of Quebec, Hydro-Quebec and Northern Quebec Cree communities in response to the La Grande Hydroelectric Complex in Quebec, Canada, an information campaign was undertaken to communicate information about mercury (e.g., fish consumption advisories) to Cree in the area [2,3]. In 1997, Chevalier et al. [2] reported data on Cree living in Northern Quebec and exposed to fish influenced by the La Grande Reservoir complex. What was seen was that mercury concentration in hair (a marker of mercury exposure) in men and women (40 years old and older) and women between 15 and 39 years of age decreased from 1988 to 1993/94 and this decrease was attributed to a reduction in fish consumption (and/or a switch of the diet from piscivorous fish to non-piscivorous). For example, women identified as trappers between 15 and 39 years of age had a maximum hair mercury concentration of 25.3 mg/kg in 1988 (n = 96) but 10.1 mg/kg in 1993/94 (n = 96). Men and women also identified as trappers and older than 40 years of age had a maximum hair mercury concentration of 85.6 mg/kg in 1988 (n = 361) but 42.2 mg/kg in 1993/94 (n = 361). The information campaign likely leading to the reduction in fish consumption was multifaceted and information about consumption advisories was presented in communities and to subsistence fishers in a number of ways including booklets, maps, videos and placemats [3]. Information was also presented in three languages: Cree, French and English [3].

Jardine et al. [4] completed a study of health perceptions in northern Aboriginal communities from the Northwest Territories and Newfoundland and Labrador, Canada, and determined that participants appeared to understand the nature and magnitude of the risks to their health associated with lifestyle and basic physical and environmental risks. Participants said that they had received, comprehended and accepted information on these risks, however, their behaviors associated with the lifestyle risks were not modified or changed as a result of this health messaging. Communities have not significantly changed their diet to minimize contaminant exposure through the consumption of traditional food species higher in some organochlorine and metal contaminants and this could be potentially attributed to the ineffective nature of the current messaging strategy. Communities perceive that much of the current messaging is "fear based" health messaging and in the communities federal government agencies were not identified as being a key source of health information. Federal sources were also rated "low" in terms of trust. Elders were rated as a trusted source of information; however, many elders are often not sufficiently informed to be able to provide health information [4].

In a "point of view memo" written by the Contaminants Committee, Public Health Department, Cree Board of Health and Social Services of James Bay (CBHSSJB)[5] in response to hearings regarding the EM1A/Rupert River Diversion Project Environmental Review, it was suggested that fish consumption advisories recommend a pattern of fish consumption very different from traditional consumption patterns. The consumption patterns recommended were not necessarily feasible to follow. It was also suggested that advisories can lead to cultural loss since many family activities are organized around fishing and fish eating. The CBHSSJB acknowledged that contaminants were a problem, but suggest that the most important problems facing the Miyupimaatisiiwin of the Iiyiyiu Aschii population were obesity, dependencies and psychosocial problems, all of which can be managed by promoting traditional activities without sending the double and ambiguous message that it may be dangerous to eat fish [5]. CBHSSJB would like to promote traditional activities rather than impose advisories and suggested that monitoring susceptible receptors (i.e., pregnant women) who consume fish be given required information to make appropriate decisions. CBHSSJB notes that the state of Alaska has decided not to implement fish consumption advisories suggested for national application by the US EPA since the most commonly consumed fish in Alaska are already naturally low in mercury.

Fish consumption advisories in the Great Lakes area (Canada and United States) have been issued as a result of concerns associated with the health effects of ingesting fish contaminated by pollutants (e.g., mercury, PCBs). Imm et al. [6] completed a survey of anglers in the Great Lakes area of the United States and reported that approximately half of adults who consumed fish from the Great Lakes were aware of the health advisories that had been issued by the health department in their home state and that any advisory awareness was associated with annual fish consumption rates. Historical confusion regarding consumption advisories in the Great Lakes area stemmed from the fact that until the early 1990s, sport-fish consumption advisories were state-specific which led to confusion because neighboring states often provided different advice for the same shared water body. With consistent communication of the advisories, compliance increased; however, the least popular recommendation, the restriction on the amount of fish that should be eaten in a given time period, only had a compliance level of 52%. Despite an outreach program designed to inform pregnant women of the local fish consumption advisories in Wisconsin, very few of the 20% of the survey population who consumed sport fish were aware of any consumption advisories and 67% were completely unaware. However, once made aware of the advisory, 15% ate less fish and 11% ate different types of fish [7].

In order to assess the probability of fish consumption in instances when anglers were aware of consumption advisories, Jakus et al. [8] completed a Bayesian statistical analysis on data from 20 advisories issued for the Chesapeake Bay area of the United States. The mean probability that people aware of advisories were still likely to consume fish P(Consume|Aware) was roughly 49%. The mean probability that people not aware of advisories being still likely to consume fish (P(Consume|Not Aware)) was 67%. Once made aware of the fish consumption advisories, between 13% and 25% of those surveyed reported not eating any fish from contaminated waters; 23% to 26% reported changing the species targeted for consumption; and 15% to 54% reported adjusting overall fish consumption. The data and the findings presented in the report supported the supposition that fish consumption advisories cause anglers to choose other locations to fish and take fewer overall fishing trips during any given time period. Jakus' statistics correspond to compliance rates with fish consumption advisories as seen in the Great Lakes.

### Impacts of Traditional Food Consumption Advisories with Respect to Broader Changes in Diet

A position paper of the Dietitians of Canada [9], examining food security and First Nations communities, confirms that a diet rich in traditional foods has been demonstrated to be more nutritious than one of "market foods" available in the North, especially in isolated First Nations' communities. Food security, however, can be undermined by concerns about concentrations of contaminants, including metals and organochlorines in traditionally harvested resources including fish, marine mammals and game. The loss or decline in traditional land use by First Nations including participation in hunting, gathering, and fishing activities in many northern Aboriginal communities has created and increased dependence on foods both shipped from the South and commercially available [9].

Concerns about the safety of food sources and the precipitated changes in diet are further demonstrated by the issues affecting Coast Salish Communities on Vancouver Island. These communities, when questioned about food security, listed environmental concerns and food safety as reasons for food insecurity. Historically the Coast Salish collected abundant supplies of shellfish from the coastal and inlet areas (up to 16 species of fish were consumed by the communities). The "red tide" and algal blooms contributing to paralytic shellfish poisoning (PSP) and the advisories against inlet shellfish collection have significantly reduced the abundance of shellfish available for these communities [10]. The acute effects of PSP ensure compliance with consumption advisories.

While the contamination of the Arctic marine food chain is a known scientific phenomenon, Duhaime et al. [11] reported that only 14% of Inuit have altered their lifestyle or changed their habits after having heard about the risk of the presence of PCBs in the food chain, and in the breast milk of Inuit. This resistance to change is perhaps reflective or indicative of the integration of traditional foods into the social, cultural, and economic life of the Inuit of the Canadian North. In northern communities traditional food is typically preferred over imported meats and foods. These imported foods are also very costly and are a poor quality alternative to traditional fish and game known to be rich in micronutrients and protein [12]. Duhaime et al. [11] examined the economic impact of the Nunavik health authorities' objective to encourage consumption advisories designed so that the intake of PCBs in certain foods was reduced. The Nunavik Health Authority (NHA) suggested that in order to avoid or reduce contamination, people should shift towards foods with lower known levels of contamination or increase consumption of other foods with special health properties (e.g., foods high in key nutrients like omega-3-fatty acids). Results of the Duhaime et al. [11] study suggest that both the household budgets of the Inuit in Nunavik, and the regional economy are not expected to be significantly affected by the replacement of contaminated food with the purchase of store-bought meat or with the replacement of one traditional food with another known to be less contaminated. The conclusions indicated that the NHA's suggestions of substituting contaminated food or increasing consumption of other foods with special health properties would not result in economic constraints for Inuit. Duhaime et al. [11] highlight two important detractors from consumption advisories issued for traditional foods, even in situations causing exposure to contaminants; 1) traditional foods are a vital source of essential nutrients for the Inuit and provide health benefits despite the level of contaminants they contain and 2) traditional foods are integral to the social, cultural and economic life of populations and communities in the North as harvesting, sharing processing and eating traditional foods are key components to the cultural spiritual and social life of both individuals and the communities.

While consumption advisories can result in a reduction in contaminant load, they can also result in a reduction on reliance of traditional foods and subsequently lead to a reduction in the intake of beneficial nutrients. Traditional foods are an integral component of good health among Aboriginal peoples. The social, cultural, spiritual, nutritional and economic benefits of these foods and their preparation, procurement and consumption are important in the maintenance of First Nations culture as indigenous relationships to the land are based in cultural practices [10]. Traditional foods can also contribute significantly more protein, iron, vitamin A, folic acid and zinc to the diets of First Nation consumers than southern/market foods [10,13]. Moreover, increases in obesity, diabetes and cardiovascular disease have been linked to a shift away from traditional foods [14].

### Impacts of Traditional Food Consumption Advisories with Respect to Loss of Confidence

Fall and Field [15] reported that prior to the oil spill from the Exxon Valdez typical fish use values ranged from 70-275 kg/year with up to 19 species being harvested. In 1990, the year after the spill, subsistence harvesting declined between 31 and 77% in 10 of 15 Coastal Alaskan First Nation communities compared to pre-spill values. Resource sharing patterns also changed and the number of species harvested declined from 19 to 10, with subsistence harvesters reporting caution and refraining from harvesting or using traditional foods for fear of resource poisoning. Three years after the spill the harvested quantities had rebounded but were still lower than pre-spill values indicating continued concerns in some communities. Some households reportedly had returned to subsistence consumption for economic and food security reasons. Prior to the spill, subsistence harvesting by First Nations along the West Coast of B.C and Alaska was widespread with almost every household using, harvesting and participating in the non-commercial exchange of wild foods. Although the harvests were seasonal and based on resource availability, the subsistence harvests were diverse and the subsistence activities were organized as kinship or family activities with foods being shared with relatives and elders and also extending to community members in need. Differences in harvest composition also occurred after the spill; prior to the spill marine mammals comprised 37% of the harvest but only 6% after the spill. The values for fish were 38% pre-spill and 74% post-spill with the differences in fish consumption patterns largely linked to the finding that fish were generally free of oil contamination and were safe to eat. First Nation communities expressed concern about the long term effects of using subsistence food and had a perception of continued low populations of some resources. Many hunters and harvesters continued to travel outside of the Prince William Sound area to harvest for the purpose of either avoiding the oiled areas or because of scarce resources [15].

In December 1996 the Alberta Provincial Health Officer issued a public health notice advising against eating wild game taken from the Swan Hills area after an uncontrolled release from the Special Waste Treatment Centre in Swan Hills, Alberta. Precautionary measures included a consumption advisory to refrain from eating wild game caught from within a 30 km radius of the plant [16]. This advisory was downgraded in May, 1997 to limit the quantity of wild game consumed from within 30 km of the plant to 370 grams per month. As a result of the wild game consumption advisory, First Nations local to the Swan Hills area have lost confidence in the quality of moose harvested in the area. Roughly 80% of the First Nation hunters changed their harvesting locations to avoid the contaminated area in the Swan Hills, 10% stopped hunting and eating moose altogether and 10% did not change their hunting practices. Still today when moose is offered at community gatherings, the source is often questioned prior to acceptance [17].

In the 2006 dietary study conducted by Furgal et al. [18] to determine the effectiveness of risk communication messaging in informing Arctic residents of the potential presence of contaminants in their food, the majority of respondents said that they had heard about contaminants in their region. However, in cases where it was documented that traditional food was not eaten, safety and food contamination were not often given as the reasons some traditional foods were not consumed. In general, most people indicated that they knew of no specific groups who should avoid certain county foods and no one reported that women of child-bearing age or children should avoid traditional foods. Of the demographic groups included in the survey, hunters were the most likely to report having been informed of consumption advisories. Women of childbearing age who choose foods for their families and who theoretically should have the most information about contaminant levels in different traditional foods and the need to avoid certain foods, were the least aware of the advisories. More than half of the study respondents indicated that if their traditional food were contaminated, they would not change their food habits, assuming that they could avoid any problems by visual checking, extra cooking, or hospital check-ups [19].

Boult [20] examined food insecurity in Arctic communities and established that a number of factors have influenced both harvesting practices and the consumption of traditional food. The study showed a decrease in the amount of traditional food that was included in the local diet. While climate change may have a negative impact on harvesting activities, concerns have also been raised about environmental pollutants, the contamination of the Arctic food chain, and the consumption of traditional foods.

## Conclusions

We identified that there are both positive and negative aspects to the issuance of traditional food consumption advisories. Studies have shown that when communicated effectively and clearly, and when community members are involved in the process, consumption advisories can result in a decrease in contaminant load in people. Conversely, it appears that traditional food consumption advisories have the potential to lead to cultural loss of the Peoples for whom the advisories were developed in the first place. Traditional food consumption advisories have been linked to a certain amount of social, psychological, nutritional, economic and lifestyle disruption and in some cases, communities have decided to ignore traditional food consumption advisories opting to continue with traditional lifestyles believing that the benefits of doing so (e.g., health benefits, cultural benefits) outweigh the risk of following advisories. Indeed, some have argued that the effects of consumption advisories can be greater than the affect of exposure to the contaminants advisories were meant to protect against [21]. These variables need to be recognized during the development and implementation/communication of consumption advisories in order to ensure a balance between human health, maintenance of cultures and industrial activity.

## Abbreviations

CBHSSJB: Contaminants Committee, Public Health Department, Cree Board of Health and Social Services of James Bay; NHA: Nunavik Health Authority; PCB: polychlorinated biphenyl; US EPA: United States Environmental Protection Agency

## Competing interests

The authors declare that they have no competing interests.

## Authors' contributions

CM and LDK both researched and wrote the manuscript. Both authors read and approved the final version.
